# Transient receptor potential ankyrin1 channel is endogenously expressed in T cells and is involved in immune functions

**DOI:** 10.1042/BSR20191437

**Published:** 2019-09-20

**Authors:** Subhransu Sekhar Sahoo, Rakesh Kumar Majhi, Ankit Tiwari, Tusar Acharya, P. Sanjai Kumar, Somdatta Saha, Abhishek Kumar, Chandan Goswami, Subhasis Chattopadhyay

**Affiliations:** 1 School of Biological Sciences, National Institute of Science Education and Research, HBNI, Bhubaneswar, Jatni, Khurda 752050, Odisha, India; 2 Institute of Bioinformatics, International Technology Park, Bangaluru 560066, India; 3 Manipal Academy of Higher Education (MAHE), Manipal 576104, Karnataka, India

**Keywords:** Ca2+-influx, immune regulation, Immunity, T-cells, TRPA1

## Abstract

Transient receptor potential channel subfamily A member 1 (TRPA1) is a non-selective cationic channel, identified initially as a cold sensory receptor. TRPA1 responds to diverse exogenous and endogenous stimuli associated with pain and inflammation. However, the information on the role of TRPA1 toward T-cell responses remains scanty. *In silico* data suggest that TRPA1 can play an important role in the T-cell activation process. In this work, we explored the endogenous expression of TRPA1 and its function in T cells. By reverse transcription polymerase chain reaction (RT-PCR), confocal microscopy and flow cytometry, we demonstrated that TRPA1 is endogenously expressed in primary murine splenic T cells as well as in primary human T cells. TRPA1 is primarily located at the cell surface. TRPA1-specific activator namely allyl isothiocyanate (AITC) increases intracellular calcium ion (Ca^2+^) levels while two different inhibitors namely A-967079 as well as HC-030031 reduce intracellular Ca^2+^ levels in T cells; TRPA1 inhibition also reduces TCR-mediated calcium influx. TRPA1 expression was found to be increased during αCD3/αCD28 (TCR) or Concanavalin A (ConA)-driven stimulation in T cells. TRPA1-specific inhibitor treatment prevented induction of cluster of differentiation 25 (CD25), cluster of differentiation 69 (CD69) in ConA/TCR stimulated T cells and secretion of cytokines like tumor necrosis factor (TNF), interferon γ (IFN-γ), and interleukin 2 (IL-2) suggesting that endogenous activity of TRPA1 may be involved in T-cell activation. Collectively these results may have implication in T cell-mediated responses and indicate possible role of TRPA1 in immunological disorders.

## Introduction

Transient receptor potential cation channel subfamily A member 1 (TRPA1) is the only member of the mammalian ‘ankyrin’ type subfamily of TRP channels [1]. TRPA1 is a calcium ion (Ca^2+^)-permeable non-selective cation channel that is expressed in a set of nociceptive/thermo-receptive neurons that can detect noxious cold temperatures below 17°C. It is often co-expressed with the heat-sensitive channel, transient receptor potential cation channel subfamily transient receptor potential vanilloid1 (TRPV1) rather than with the cold-sensitive channel TRPM8. Initial reports suggested that the large influx of Ca^2+^ at lower temperatures (such as at 10°C) is primarily due to the direct activation of this channel by cold stimuli [2,3]. Apart from low temperature as a stimulus, TRPA1 also acts as a nociceptive receptor that detects noxious chemicals that can cause tissue damage. TRPA1 also acts as a mediator of inflammatory pain generated by either noxious cold or chemical irritants [4,5].

Ca^2+^ channels are an integral part of T-cell activation, differentiation, and formation of an immunological synapse between mature CD4^+^ T cells and Antigen-Presenting Cells (APC), and even exocytosis of vesicles in cytotoxic T cells. It also influences cytokine release patterns which in turn affect T-cell functions like the development of anergy, maturation, and differentiation of naïve T cells into Th1 and Th2 cells [6]. In agreement with these reports, abnormality in Ca^2+^-signaling results in several immunological disorders such as SCID and Wiskott–Aldrich syndrome (WAS) [6]. T-cell motility is also regulated by Ca^2+^ in consortium with protein kinase C (PKC) which regulates the rearrangement of actin cytoskeleton in them. Activation of several transcription factors such as NFAT, NF-κB, calmodulin-dependent kinase, JNK, and others are known to be involved in T-cell regulation and require Ca^2+^ [7]. Moreover, recently a strategic usage of cation channel blockers toward T-cell activation, cytokine secretion, and proliferation has been demonstrated [8]. Transient receptor potential (TRP) channels act as Ca^2+^-permeable channels and thereby regulate Ca^2+^ homeostasis. So far, few members of the TRP family have been identified, by us and other groups, that are endogenously expressed in T cells and regulate T-cell functions [9,10].

Endogenous inflammatory agents such as Reactive Oxygen Species (ROS) induce the production of 4-hydroxy-2-nonenal (HNE) that directly activates TRPA1 [9]. Nitrated fatty acids and prostaglandins like 15d-PGJ2 are released at the site of inflammation, and such compounds can also directly activate TRPA1 [10,11]. It is also known that lymphoma and inflammation associated itch is mediated by a Th2 cell-derived cytokine, interleukin (IL)-31 (IL-31), that directly interacts with its receptor IL-31RA located on TRPV1^+^/TRPA1^+^ sensory nerves in skin [12]. These compounds can also play a role in T-cell activation. In addition, lipopolysaccharide (LPS, a noxious by-product of Gram-negative bacteria) activates TRPA1 via a TLR4-independent mechanism and thereby generates a rapid nociceptive response and neurogenic inflammation [13]. In this work, we have probed the expression, localization of TRPA1 channel in T cells and also characterized the function of TRPA1 toward T-cell activation.

## Materials and methods

### Bioinformatics analysis

TRPA1 gene input was made into Search Tool for the Retrieval of Interacting Genes/Proteins (STRING 10) [14] web tool for exploring protein–protein interactions with the confidence score higher than 0.7 with the top interacting partners; Kyoto Encyclopaedia of Genes and Genomes (KEGG) and Gene Ontology annotations were deduced for TRPA1 interacting partners using g:profiler webserver [15].

### Reagents

The TRPA1 channel modulatory drugs allyl isothiocyanate (AITC, activator), A-967079 (inhibitor), and HC-030031 (another inhibitor) were obtained from Sigma–Aldrich (St. Louis, MO, U.S.A.). Concanavalin A (ConA) was purchased from HiMedia (India). Knockout validated rabbit polyclonal antibody against the first extracellular loop (747–760 aa) of hTRPA1 and its specific blocking peptide (NSTGIINETSDHSE) were purchased from Alomone Laboratories (Jerusalem, Israel; Cat. no: ACC-037). The other rabbit polyclonal antibody against the N-terminus of TRPA1 was procured from Novus Biologicals (Centennial, CO, U.S.A.; Cat. no: NB110-40763). The Ca^2+^-sensitive dye Fluo-4AM was procured from Molecular Probes (Eugene, OR, U.S.A.). Calcium chelating agents BAPTA-AM (1,2-bis(2-aminophenoxy)ethane-N,N,N′,N′-tetraacetic acid tetrakis (acetoxymethyl ester)) and EGTA (ethylene glycol-bis(β-aminoethyl ether)-*N*,*N*,*N*′,*N*′-tetraacetic acid) were procured from Sigma (St. Louis, MO, U.S.A.). TRIzol was purchased from Life Technologies (Carlsbad, CA, U.S.A.). Verso cDNA synthesis kit was obtained from Thermo Scientific (Waltham, MA, U.S.A.). SYBR Green for reverse transcription polymerase chain reaction (RT-PCR) was procured from Life Technologies (Carlsbad, CA, U.S.A.). The RT-PCR primers for TRPA1 and GAPDH were obtained from IDT (Coralville, IO, U.S.A.). Anti-mouse cluster of differentiation 25 (CD25)-PE, cluster of differentiation 69 (CD69)-PE, and anti-human CD3-PE as well as functional grade (azide-free) anti-CD3 and anti-CD28 mAbs were obtained from BD Biosciences (San Jose, CA, U.S.A.). CD3/CD28 T-cell activation beads were purchased from Thermo Fisher (Gibco). The CD90.2-APC antibody was from Tonbo Biosciences (San Diego, CA, U.S.A.). The sequences of the primers used in the study are mentioned below:

TRPA1 Forward: 5′- GTC CAG GGC GTT GTC TAT CG - 3′
TRPA1 Reverse: 5′- CGT GAT GCA GAG GAC AGA AT - 3′
GAPDH Forward: 5′- CCG CAT CTT CTT GTG CAG TG- 3′
GAPDH Reverse: 5′- CCC AAT ACG GCC AAA TCC GT- 3′.

### Isolation and culture of T cells

T cells were isolated and cultured as described previously [16]. Briefly, murine spleen cells were obtained from 6- to 8-week-old BALB/c mice as per the approval of the Institutional Animal Ethics Committee (IAEC protocol no. NISER/SBS/IAEC/AH-39). Single cell suspension was made by passing the suspended splenocytes through a 70-μm cell strainer. T cells were purified from the non-adherent splenocyte population by using BD IMag™ Mouse T Lymphocyte Enrichment Set – DM according to the manufacturer’s instructions. The isolated cells were cultured in a 24-well polystyrene cell culture plate (3 × 10^6^ cells/well) with RPMI (PAN Biotech, Aidenbach, Germany) supplemented with 10% FBS (PAN Biotech). The percentage purity of the purified T cells was above 95% in each case. All the experiments were performed at approximately 36 h after plating the cells as most of the primary T cells were found to be activated during 36–48 h after ConA or TCR treatment (data not shown). Primary murine T cells were activated with either a combination of plate-bound α-CD3 (2 μg/ml) and soluble α-CD28 (2 μg/ml), or with ConA (4 μg/ml) alone for 36 h before experiments. Similarly, human peripheral blood mononuclear cell (hPBMC)-derived T cells were purified by Human T cell isolation kit from Invitrogen (Invitrogen Dynal AS, Oslo, Norway) according to manufacturer’s instructions. Treatment of T cells was carried out with selective TRPA1 activator AITC (100 μM) and inhibitor A-967079 (100 μM) with or without ConA or α-CD3/CD28.

### RNA isolation and RT-PCR

Approximately 5 × 10^6^ T cells were used for RNA isolation. For positive control, spinal cord tissue from mice was used. RNA isolation was done using TRIzol reagent according to the manufacturer’s instructions. Nanodrop readings were taken and 1 μg of RNA was converted into cDNA using verso cDNA synthesis kit as per the mentioned protocol. RT-PCRs were performed for TRPA1 and GAPDH in ABI7500 system (Applied Biosystems, Foster City, CA, U.S.A.) using 2× SYBR Green Mix following the reaction gene expression was visualized in 1% agarose gel.

### Flow cytometry

Flow cytometry analysis of T cells was performed as described previously [16]. For probing TRPA1 expression, cells were stained with the TRPA1-specific antibody mentioned before and subsequently flow cytometric analysis was performed as described previously [16,17]. For evaluating the profile of immune markers, mouse T cells were incubated with anti-CD25-PE, CD69-PE and CD90.2-APC mAbs dissolved in FACS buffer (1× PBS, 1% BSA, and 0.05% sodium azide) for 30 min on ice and then washed further. Stained cells were washed twice with the same FACS buffer before the line-gated acquisition of approximately 10000 cells. hPBMC-derived T cells were stained with anti-human CD3 antibody and were acquired with FACS Calibur (BD Biosciences). Data were analyzed using CellQuest Pro software (BD Biosciences). The percentages of cells expressing the markers are represented in dot-plots while the MFI values represent the expression levels of the markers per cell.

### Immunofluorescence analysis and microscopy

Immunofluorescence analysis of T cells was performed as described previously [16]. For immunocytochemical analysis, immediately after harvesting, T cells were diluted in PBS and fixed with paraformaldehyde (final concentration 2%). After fixing the cells with paraformaldehyde, immunostaining was done by two procedures: in first case cell permeabilization was not performed as the antibody detecting TRPA1 recognizes an extracellular region of TRPA1. In other cases, the cells were permeabilized with 0.1% Triton X-100 in PBS (5 min). Subsequently, the cells were blocked with 5% BSA for 1 h. The primary antibody was used at 1:400 dilution. In some experiments, blocking peptides were used to confirm the specificity of the immunoreactivity. The ratio of blocking peptides with specific antibody was 1:1 (in concentration). Another rabbit polyclonal antibody (procured from Novus Biologicals) detecting epitope present in the N-terminal cytoplasmic domain of TRPA1 was used (1:1000 dilution) in some experiments to confirm the endogenous expression of TRPA1 in T cells. All primary antibodies were incubated overnight at 4°C in PBST buffer (PBS supplemented with 0.1% Tween-20). AlexaFluor-488 labeled anti-rabbit antibody (Molecular Probes) was used as secondary antibody and at 1:1000 dilutions. All images were acquired on a confocal laser scanning microscope (LSM-780, Zeiss) with a 63× objective and analyzed with the Zeiss LSM image examiner software followed by compilation using Adobe Photoshop.

### Ca^2+^ imaging

Ca^2+^ imaging of primary murine splenic T cells was performed as described previously with minor modifications [16,18]. In brief, primary murine splenic T cells were loaded with Ca^2+^- sensitive dye (Fluo-4 AM, 2 μM for 30 min). Ca^2+^-chelation experiment was performed by treating the cells with 5 μM BAPTA-AM for 1 h and extracellular Ca^2+^ was chelated with 5 μM EGTA for 1 h. The cell suspension was added to the live cell chamber for Ca^2+^ imaging and images were acquired at every 5-s intervals. The cells were stimulated with specific agonists alone or in a combination of agonists and antagonists and CD3/CD28 T-cell activation beads as described. Fluo-4 AM signal was acquired using a Zeiss microscope (LSM 780) and Olympus microscope (FV3000) and with the same settings. The images were analyzed using LSM software and Fiji. The intensities specific for Ca^2+^-loaded Fluo-4 are represented in artificial rainbow color with a pseudo scale (red indicating the highest level of Ca^2+^ and blue indicating the lowest levels of Ca^2+^). For quantification of the changes in the intracellular Ca^2+^ levels, fluorescence intensity of T cells present in view field was measured before and just after adding the drugs. Such values were plotted for relative changes.

### Enzyme-linked immunosorbent assay

Supernatants from the respective experiments were collected and stored at −80°C. Enzyme-linked immunosorbent assay (ELISA) for different T-cell effector cytokines tumor necrosis factor (TNF), IL-2, and interferon-γ (IFNγ) were performed using BD Biosciences Sandwich ELISA kits (San Jose, CA, U.S.A.) as per the manufacturer’s instructions. The readings were acquired using a microplate reader (Bio-Rad iMARK) at 450 nm.

### Statistical tests

The primary data were imported into R software for statistical analysis. The ANOVA test was performed for dataset comprising more than two experimental groups. To check the reliability and significance. Student’s *t* test was performed in GraphPad Prism 7 to derive significance of the calcium imaging data with two experimental groups. The *P*-value of <0.05 was considered statistically significant. Data presented here are representative of three independent experiments. The significance values are as follows: ****, *P*≤0.0001; ***, *P* between 0 and 0.001; **, *P* between 0.001 and 0.01; *, *P* between 0.01 and 0.05; ns, *P* above 0.05.

## Results

### Gene set enrichment analysis reveals that TRPA1 has immune function

**Figure 1:**
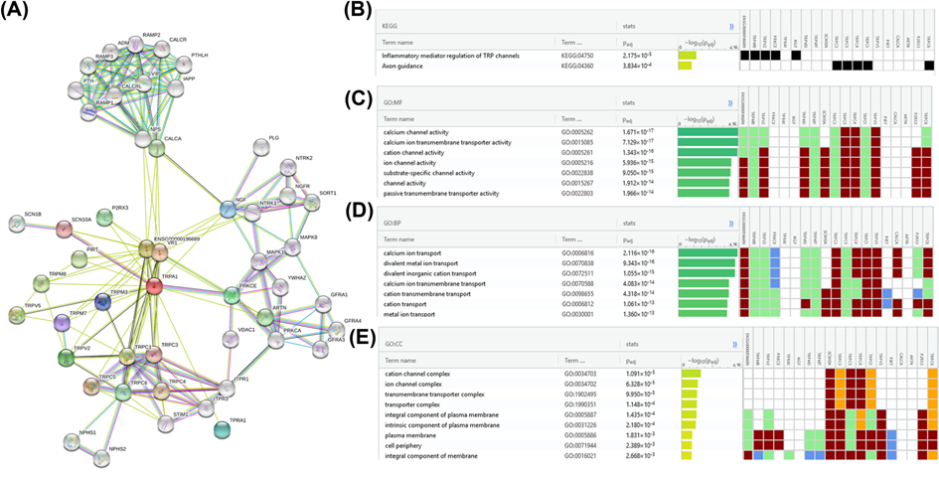
Possible involvement of TRPA1 in immuno-functions based on protein interaction data (**A**) Overview of protein–protein interaction partners of TRPA1. The interaction network suggests that TRPA1 may be involved in inflammatory processes based on KEGG (**B**) and GO annotations MF (**C**), BP (**D**) and CC (**E**). Abbreviations: BP, biological process; CC, cellular component; MF, molecular function.

Protein–protein interaction patterns of TRPA1 were examined using STRING11 [14] with confidence cut-off score (>0.7) (Figure 1A). These proteins interacting with TRPA1 were evaluated for their roles using gene set enrichment analysis via g: Profiler webserver [15]. This computational analyses suggests that TRPA1 is potentially associated with immune function associated processes along with typical function as of ion channels (Figure 1B–E). This indicates that TRPA1 might possibly be involved in regulation of immune system. Hence, this imposed further experimental evaluation.

### TRPA1 is expressed endogenously in primary murine and human T cells

**Figure 2:**
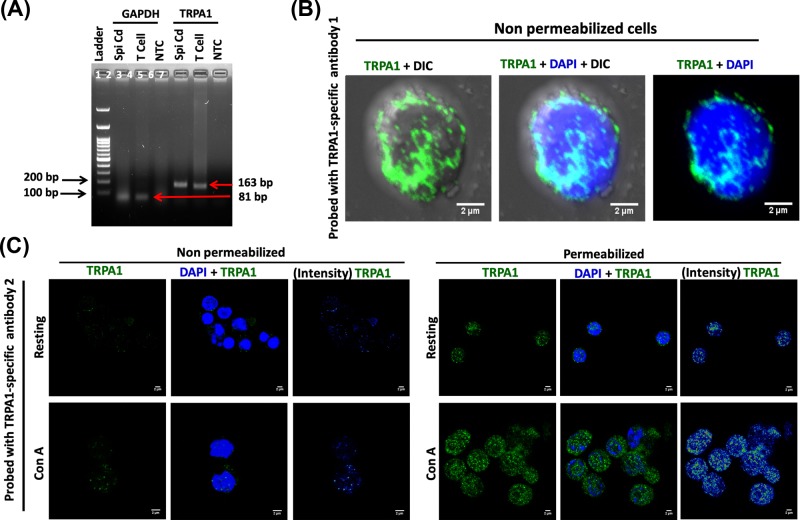
Endogenous expression of TRPA1 primary murine T cells (**A**) RT-PCR for TRPA1 and GAPDH from mRNA isolated from T cell. Total mRNA isolated from mouse spinal cord is used as a positive control and no-template control (NTC) is used as negative control. (**B**) Immunolocalization of TRPA1 in the surface of unpermeabilized T cells. (**C**) Immunodetection of TRPA1 in T cell by using another antibody recognizing the epitope present in the N-terminal cytoplasmic domain. In permeabilized cells this antibody detects TRPA1 at low levels in resting condition and modest level in ConA-treated condition. This antibody does not detect TRPA1 in non-permeabilized T cells as its epitope at N-terminus is located in intracellular region.

Expression of TRPA1 at mRNA level in T cells was confirmed by RT-PCR (Figure 2A). The surface expression of specific ion channels is critical for signaling events. Therefore we used a specific antibody (from Alomone labs) for which the epitope is present at the extracellular loop-1 of TRPA1 (i.e., present outside the cell surface). This antibody allowed us to probe the surface expression of TRPA1 (in unpermeabilized cells) and as well as total TRPA1 expression (in Triton X-100-permeabilized cells). This antibody detected endogenous TRPA1 signal at the surface of unpermealized T cells (Figure 2B). To confirm the endogenous expression of TRPA1 in T cell, we used another antibody (Novus Biologicals) raised against epitope present in the N-terminal cytoplasmic domain of TRPA1. This antibody detects TRPA1 modestly in resting T cells and strongly in ConA (a lectin that acts as a mitogen and results in T-cell activation) activated T cells, but after permeabilization (Figure 2C, right-hand side). This antibody does not detect TRPA1 in unpermeabilized T cells, indicating specificity of the antibody (Figure 2C, left-hand side). Taken together, the data strongly suggest that TRPA1 is endogenously expressed in murine T cells.

Next, we probed surface as well as total expression of TRPA1 in murine T cells that are at resting (naïve) stage and/or activated with either ConA or by T-cell receptor (TCR) stimulation with α-CD3/α-CD28 antibodies [19,20].

**Figure 3:**
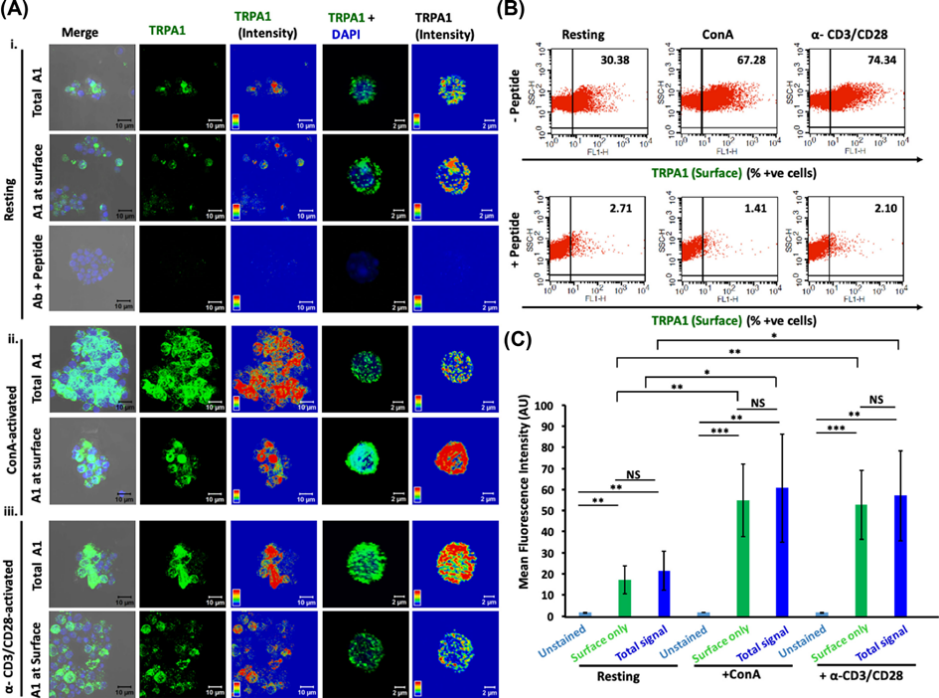
TRPA1 expression increases during murine T-cell activation (**A**) Expression of TRPA1 is increased in activated T cells compared with the resting conditions. TRPA1 present at the surface only (in non-permeabilized cells) and in whole cell (in permeabilized cells) were probed. Triton X-100 permeabilized cells when stained with TRPA1-specific antibody pre-incubated with its antigenic peptide results in loss of TRPA1 signal, indicating the specificity of the antibody. Confocal images of purified CD3^+^ murine T cells stained with antibody detecting the extracellular loop of TRPA1 are shown. Both surface level and total expression of TRPA1 in resting cells (**i**), in ConA-activated cells (**ii**), and in α-CD3/α-CD28-activated T cells (**iii**) are shown. Fluorescence intensity of the TRPA1 signal is indicated in rainbow scale (right panels). (**B**) Dot-plot values indicating the percentage of cells expressing TRPA1 at the surface. The number of TRPA1^+ve^ cells are much higher in activated conditions. This staining is completely blocked when the same antibody is pre-treated with its specific blocking peptide. (**C**) Mean fluorescence intensity determined by flow cytometry analysis reveal that ConA-activated and α-CD3/α-CD28-activated T cells have higher levels of TRPA1 than the resting T cells. The difference in TRPA1 expression level (both in surface as well in whole cell) between resting stage with ConA-activated or α-CD3/α-CD28-activated T cells are significant. The *P-*values are: ns, non-significant; *, <0.05; **, <0.01; ***,<0.001.

Confocal microscopy of unpermeabilized cells revealed that TRPA1 is endogenously expressed in resting and activated T cells as distinct clusters that are primarily located at the cell surface (Figure 3A(i)). Notably, the TRPA1 signal was blocked upon pre-incubating the antibodies with their antigenic peptide confirming the specificity of the antibody used (Figure 3A(i),B). The intracellular localization of TRPA1 was almost minimal as there was no significant difference in its expression in surface versus in whole cell (in resting conditions) (Figure 3A,C). However, all the T cells do not express TRPA1 at resting state. Flow cytometry results confirmed that the expression level of TRPA1 was increased in ConA-activated and in TCR-activated T cells (Figure 3B,C).

**Figure 4:**
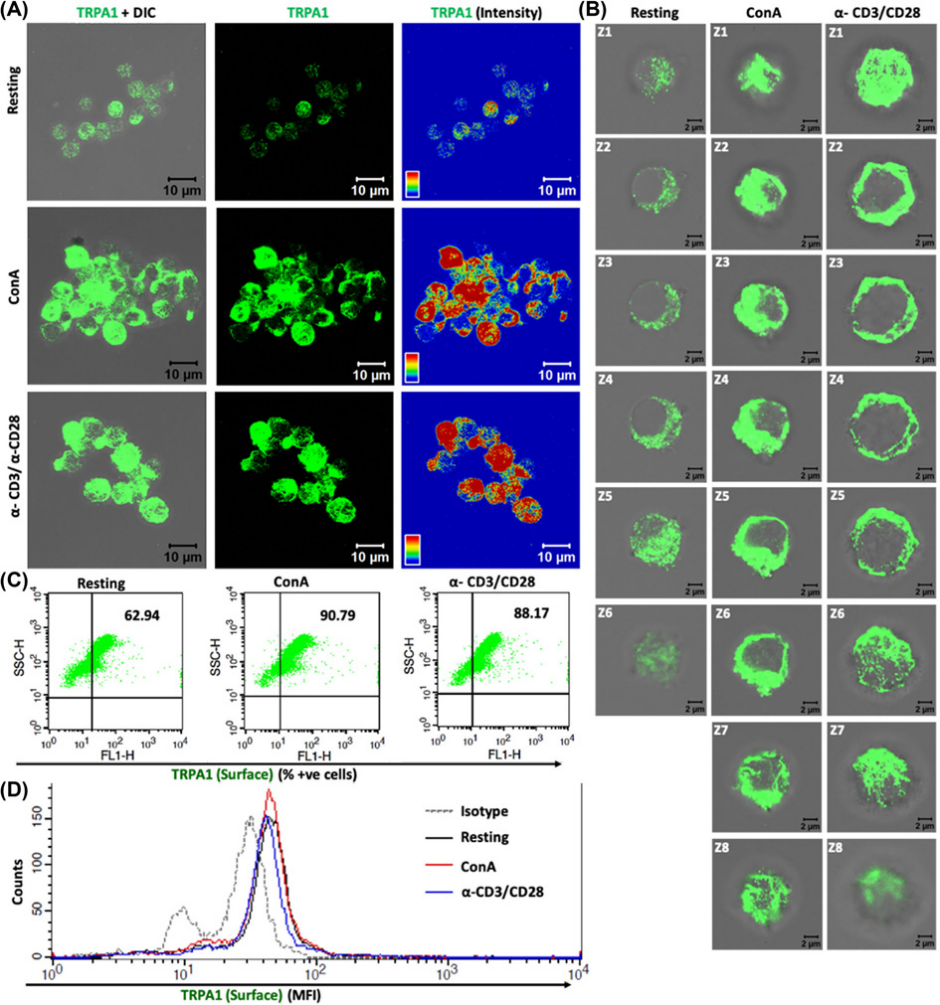
Human T cells show increased surface expression of TRPA1 upon activation (**A**) Confocal imaging revealed that TRPA1 is endogenously expressed in purified CD3^+^ human T cells and TRPA1 expression increases in activated T cells. (**B**) Optical sections (Z1–Z8) of T cells reveal that TRPA1 is present mostly at or near the cell surface both in resting and in activated conditions. (**C**) Flow cytometry dot-plot values indicate that approximately 60% of human T cells are TRPA1^+ve^ under resting conditions while approximately 90% cells are TRPA1^+ve^ in immunologically activated state. (**D**) MFI for TRPA1 of hPBMC-derived T cells in resting, ConA, and TCR stimulated cells.

Similar to murine T cells, hPBMC-derived T cells also show TCR and ConA activation driven increased expression of TRPA1 (Figure 4A). The Z-section images showed an increase in TRPA1 at the surface on T-cell activation by ConA or TCR (Figure 4B). Moreover, we have also studied the expression of TRPA1 in resting and activated hPBMC-derived T cells and found that percent positive cells for TRPA1 was increased in activated T cells as compared with resting T cell (Figure 4C). However, we did not observe any marked change in the MFI of TRPA1 in hPBMC-derived T cell in resting and activated cells (Figure 4D). These qualitative and quantitative data strongly suggest that TRPA1 is endogenously expressed in T cells and increased surface expression of TRPA1 correlates with T-cell activation process.

### TRPA1 activation induces increased Ca^2+^ level in T cells

**Figure 5:**
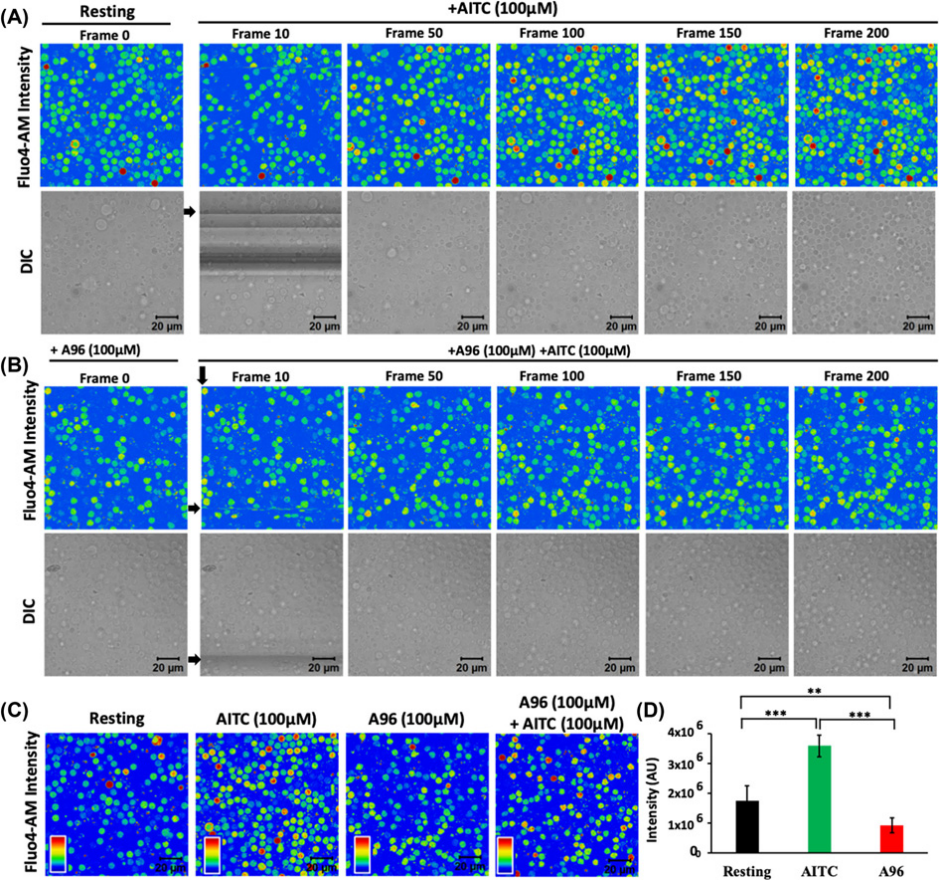
TRPA1 activation induces increase in intracellular Ca^2+^ levels in murine T cells (**A,B**) Fluorescence intensity images derived from time-series imaging of view fields containing multiple cells loaded with Ca^2+^-sensing dye Fluo 4-AM are depicted here. The time difference between each frame is 5 s. The cells were treated with different pharmacological agents at the 10th frame (F10). Activation of TRPA1 by its specific activator AITC (100 μM) causes increment in the Ca^2+^ level (A) which can be blocked by pre-incubating the cells with TRPA1-specific inhibitor A967079 (A96; 100 μM) for 30 min (B). (**C**) Fluo 4 intensity of T cells at resting stage or incubated with AITC (100 μM), A96 (100 μM), or combination of AITC (100 μM) and A96 (100 μM) for 12 h is shown. (**D**) Quantification of Fluo-4 intensity from six random fields after acquisition of 200 frames (∼1000 s) of two independent experiments is depicted. AITC (100 μM) treatment significantly increases Ca^2+^ levels, while A96 (100 μM) treatment reduces intracellular Ca^2+^ levels below that of resting T cells. The *P-*values are: ns, non-significant; *, <0.05; **, <0.01; ***, <0.001.

**Figure 6:**
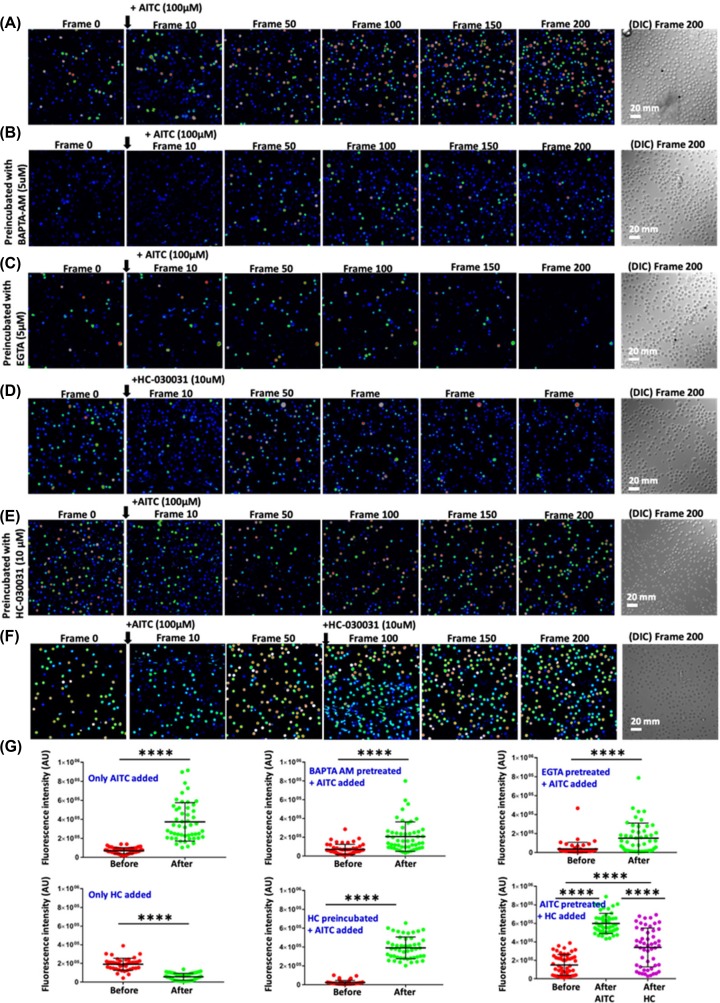
TRPA1 activation-induced increase in intracellular Ca^2+^ can be altered by Ca^2+^-chelators and channel inhibitors Fluorescence intensity images derived from time-series imaging of view fields containing multiple cells loaded with Ca^2+^-sensing dye Fluo 4-AM are depicted here. The time difference between each frame is 5 s. Untreated cells at resting stage or pre-incubated with specific Ca^2+^-chelators or TRPA1 blocker. Activation of TRPA1 by AITC at the 10th frame causes increment in the Ca^2+^ level (**A**) which can be reduced by pre-incubating the cells with BAPTA-AM (intracellular Ca^2+^-chelator) (**B**) or with extracellular Ca^2+^-chelator (EGTA) (**C**). Application of HC-030031, another inhibitor of TRPA1 results in reduced intracellular level of Ca^2+^ (**D**). Application of AITC on population preincubated with HC-030031 fails to increase the basal Ca^2+^ levels (**E**). Application of HC-030031 after AITC results in initial rise (due to activation) followed by reduction (due to inhibition) in the basal Ca^2+^ levels (**F**). In each case, the DIC image represents the number and morphology of cells in the view-field at the end of the experiments (200th frame). (**G**) Quantification of basal Ca^2+^ levels in multiple cells before and after addition of TRPA1 modulatory agents. The maximum time gap between these two measurements is ∼5 s. **** indicates *P*-value <0.0001. For more details see Supplementary Movies.

In order to explore if the TRPA1 present in T cells are functional, we performed Ca^2+^-imaging experiments (Figure 5). For that purpose, we have used purified mouse T cells loaded with Fluo-4 AM. Live cell imaging revealed that in the absence of any stimuli, there is no increase in Fluo-4 intensity in majority of the cells with respect to time. However, upon stimulation by TRPA1 activator AITC, intracellular Ca^2+^ level increases in most of the T cells (Figure 5A). This AITC-mediated increase in Ca^2+^ level was effectively blocked by TRPA1-specific inhibitors A-967079, and HC-030031 (Figures 5 and 6). Calcium chelation experiments with BAPTA-AM as well as by EGTA (present in extracellular media) suggest that TRPA1 regulates Ca^2+^influx from both extracellular and intracellular reserves (Figure 6). Similarly, the level of intracellular Ca^2+^ goes down after adding the inhibitor in activator (AITC) treated cells (Figure 6F). To confirm all these effects quantitatively, we compared the level of Ca^2+^ in T cells just before and after adding the TRPA1 modulatory drugs (Figure 6G). The analysis clearly suggests the changes in the fluorescence intensity in quick time. This confirmed that functional TRPA1 is expressed in T cells.

### TRPA1 regulates TCR-mediated Ca^2+^-influx during T-cell activation

**Figure 7:**
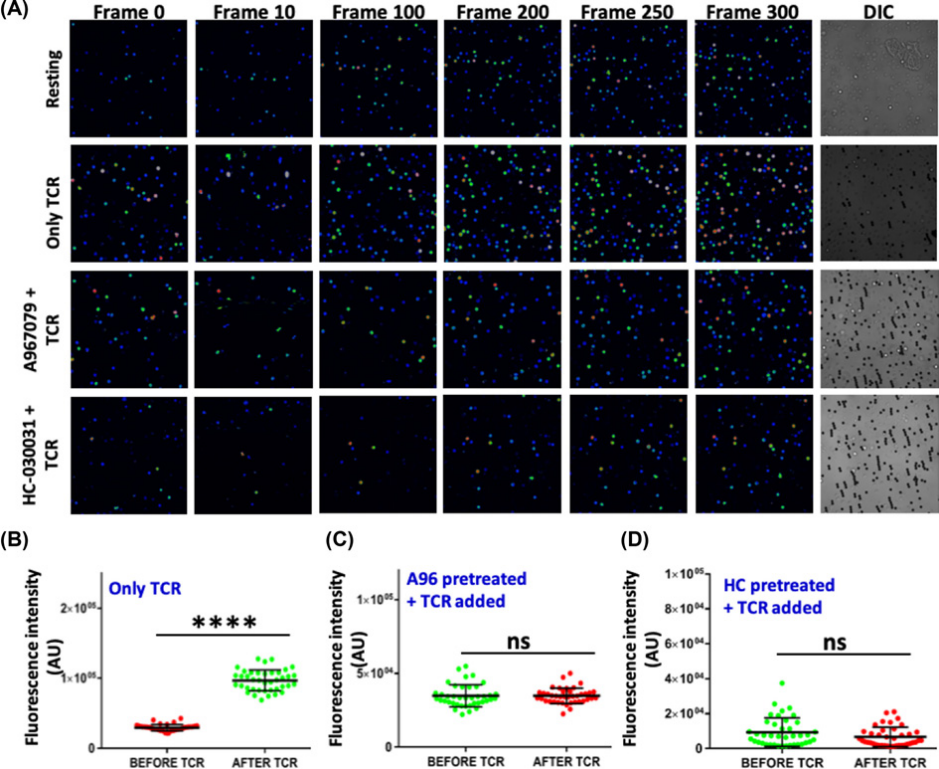
TRPA1 regulates TCR mediated Calcium influx (**A**) Representative images in rainbow scale shows addition of α-CD3/CD28 (TCR) beads elevates Ca^2+^ levels in murine T cells compared with the resting T cells. Reduced Ca^2+^ levels observed in T cells treated with TRPA1 inhibitors A967079 and HC-030031 upon TCR-mediated stimulation. (**B**) Graph showing elevated calcium levels in T-cell population after addition of TCR beads. (**C,D**) Graph shows no significant change in calcium levels in A967079 and HC-030031 treated T cells after TCR beads treatment, respectively. ****P< 0.0001.

TCR-mediated T-cell stimulation leads to increase in intracellular Ca^2+^ levels in T cells which is critical for optimum T-cell activation. As we have observed that TRPA1 activation initiates Ca^2+^ influx in T cells; hence we studied the effect of TRPA1 inhibition in Ca^2+^ influx during TCR-mediated stimulation. TCR stimulation increased intracellular Ca^2+^ levels (Figure 7A,B), while TRPA1 inhibition by A-967079 and HC-030031 significantly reduced the TCR-mediated Ca^2+^ influx in T cells (Figure 7A,C,D).

### TRPA1 is involved in ConA/TCR-mediated T-cell activation and effector cytokine secretion

**Figure 8:**
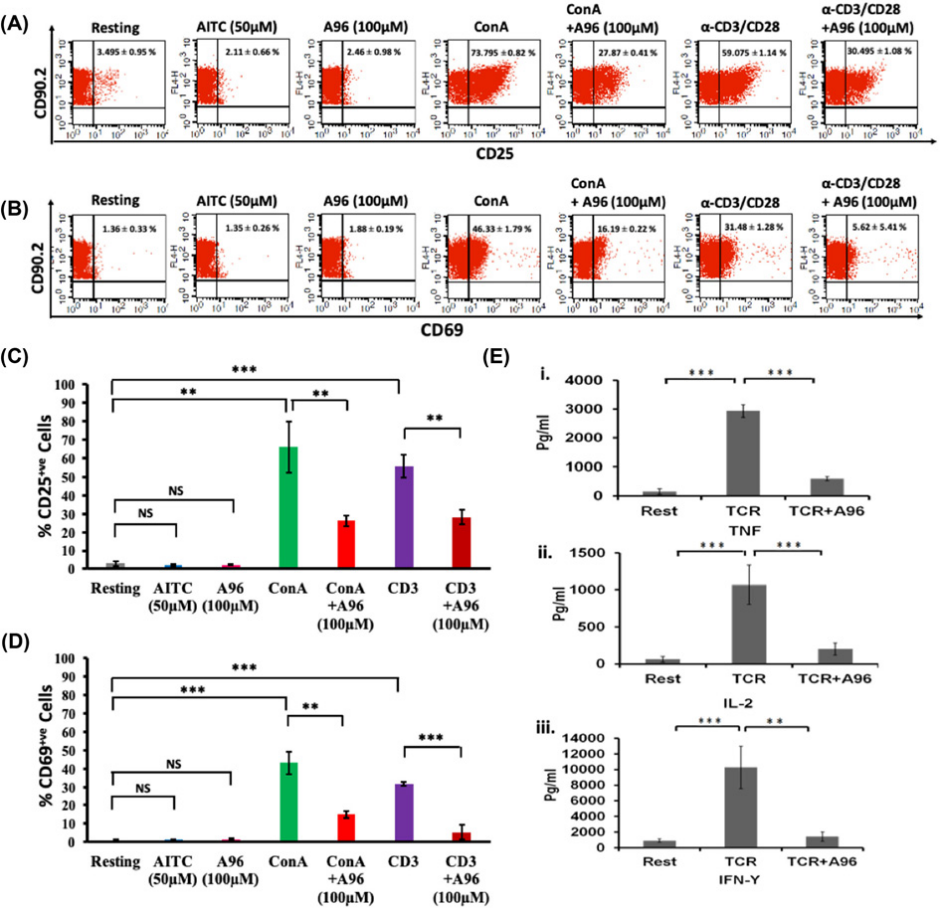
Pharmacological inhibition of endogenous TRPA1 blocks T-cell activation (**A,B**) T-cell activation markers CD25 and CD69 were analyzed by flow cytometry after incubating the cells with TRPA1 modulators for 36 h. Inhibition of TRPA1 by A96 (100 μM) reduces the ConA-mediated activation. Treatment of murine T cells with AITC (50 μM) or A96 (100 μM) alone do not increase the % of CD25^+^ cells or % of CD69^+^ cells. The average number ± SD values of CD25^+^ or CD69^+^ cells are mentioned in the upper right corner of each dot-plot. Representative dot plots of three independent experiments are shown. (**C,D**) T cells treated with A96 (100 μM) along with ConA (5 μg/ml) or plate-bound α-CD3 (2 μg/ml) and soluble α-CD28 (2 μg/ml) have reduced percentage of CD25^+^ or CD69^+^ cells. The corresponding levels/intensity of CD25 or CD69 expression (determined from MFI values) in response incubation with indicated modulators are shown (*n*=3). (**E**) Graphical bars represent the concentration (in pg/ml) of effector cytokines TNF (**i**), IL-2 (**ii**), IFNγ (**iii**) released from T cells at approximately after 36 h. Incubation of TRPA1 inhibitor A96 (100 μM) along with plate-bound α-CD3 (2 μg/ml) and soluble α-CD28 (2 μg/ml) resulted in significant reduction in the release of cytokines TNF, IL-2 and IFNγ. The *P*-values are: ns, non-significant; **, <0.01; ***, <0.001 (*n*=3 independent experiments).

In order to explore the role of TRPA1 in T-cell activation, we have activated the cells either by ConA or via TCR stimulation and probed for the expression of activation markers, namely CD25 and CD69 in the purified murine T cell (CD90^2+^) population. The expression of these markers was probed after ConA treatment with or without TRPA1 channel modulators (Figure 8). Flow cytometric evaluation revealed a shift in the T-cell population expressing CD25 upon T-cell activation (in resting condition, 3.495 ± 0.95%; in ConA-activated condition, 73.795 ± 0.82%; in TCR-activated condition, 59.075 ± 1.14%). Notably, in the presence of TRPA1 inhibitor (A-967079, 100 μM), T-cell activation by ConA or TCR was significantly inhibited (Figure 8A,C). In this condition (after treatment with both ConA and A-967079), 27.87 ± 0.41% of the cells express CD25. Similar down-regulation was seen when cells were treated with TCR in combination with A-967079, where only 30.495 ± 1.08% T cells are found to be CD25^+^.

In a similar manner, TRPA1 inhibitor (A-967079) also reduced CD69 expression. The effect of TRPA1 inhibitor on CD69 expression was also reflected by the percentage of CD69 positive cells (in resting condition, 1.36 ± 0.33%; in ConA-activated condition, 46.33 ± 1.79%; in TCR-activated 31.48 ± 1.28%) which was reduced after treating with the inhibitor (in combination of ConA and A-967079, 16.19 ± 0.22%; in TCR and A-967079 combination, 5.62 ± 5.41%) (Figure 8B,D).

T-cell activation involves an increased level of secretion of several effector cytokines like TNF, IFNγ, and IL-2. We explored the role of TRPA1 in the production of these cytokines by analyzing the culture supernatants by ELISA (Figure 8E). TCR-stimulation induced high levels of TNF (resting condition: 151.8 ± 94.88 pg/ml; TCR-mediated activated condition: 2931.98 ± 220.85 pg/ml) while inhibition of TRPA1 by A-967079 blocked the effect of these stimulators on TNF production (TCR + A-967079 combination: 597.74 ± 71.83 pg/ml) (Figure 8E). TRPA1 inhibition also blocked IL-2 secretion (resting: 62 ± 37.91 pg/ml; TCR: 1067.4 ± 267.003 pg/ml; TCR + A-967079 combination: 200.33 ± 83.25 pg/ml) (Figure 8E). Moreover IFNγ production was down-regulated by A-967079 treatment (resting: 912.83 ± 216.20 pg/ml; TCR: 10279.5 ± 2702.65 pg/ml; TCR + A-967079 combination: 1421.72 ± 599.3 pg/ml) (Figure 8E).

Taken together, the results indicate that TRPA1 is expressed in T cells and may positively regulate T-cell activation and effector cytokine release.

## Discussion

T-cell activation involves several distinct signaling events, and the influx of Ca^2+^ is vital during this process. However, so far only a few Ca^2+^ channels have been detected in T cells, whereas the identity of the major Ca^2+^ channels present in T cells is unknown. In addition, the mode of regulation of these channels and their exact role in the context of T-cell functions are mostly unknown. Although expression of TRPA1 in the peripheral sensory neurons and its involvement in the neural excitation has been widely demonstrated, limited information is currently available about the expression and role of TRPA1 in non-neuronal cells. In this work, the endogenous expression of TRPA1 in primary murine and human T cell was observed by using two different TRPA1 specific antibodies. Expression of TRPA1 was found to be mostly predominant at the surface of these cells rather than being present in intracellular regions. We also provided evidence for the functional role of TRPA1 in immune functions. Flow cytometric analysis coupled with confocal imaging conclusively suggested enhanced expression of TRPA1 (both at surface level and in total) in activated T cells as compared with resting conditions. TRPA1 activation by specific ligands leads to increased intracellular Ca^2+^ concentration in purified murine T cells, confirming that this channel is present in a functional form in resting T cells. Moreover TRPA1 inhibition down-regulated the Ca^2+^ influx during TCR-mediated T-cell stimulation.

TRPA1 can be activated by several means and also by endogenous factors. Though TRPA1 has been initially considered as a ‘cold-activated ion channel’, its true thermosensitive nature is debatable. For example, it has been shown that TRPA1 is not directly gated by cold but rather gated by increased intracellular Ca^2+^ levels as a consequence of cooling [21]. Recently TRPA1 has been demonstrated as a sensor of noxious heat (≥45°C) in association with two other heat sensors TRPV1 and TRPM3 [22]. Notably, animals where both TRPV1 and TRPA1 were knocked out, still continued to sense noxious heat, indicating that these channels are not essential and in the absence of TRPV1 and TRPA1, some other ion channel/s take/s up their function [22]. Further, it has been demonstrated that pharmacological inhibition of TRPA1 by HC030031 (100 μM) shows the same effect as TRPA1 knockout mice [22]. In these cases, at least in neuronal systems, any increase in neuronal firing or heat avoidance is observed in mice lacking TRPA1. This suggests that TRPV1 and TRPA1 act in synergistic manner, rather than TRPA1 inhibiting TRPV1 as proposed recently [23].

In this work, we demonstrated that functional TRPA1, a non-selective cation channel is expressed in the human and murine T cells. Our results suggest that TRPA1 plays an important role relevant to T-cell activation, much similar to that of two other heat sensors namely TRPV1 and TRPV4 reported by us earlier [16]. TRPA1 modulation by different endogenous factors are likely to play a major role in this process. The functional expression of TRPA1 in murine and human CD4^+^ T cells has also been shown to regulate T-cell activation and having anti-inflammatory role as reported recently [23]. However, in contrast with these findings, so far majority of the previous reports have suggested a pro-inflammatory role of TRPA1 toward immune activation and/or inflammation [24]. TRPA1 has been considered as one of the critical regulators of neurogenic inflammation and neuropeptide release [25]. It has also been reported that TRPA1 is associated with inflammation and puritogen responses in dermatitis [26]. A recent paper has shown that both TRPV1 and TRPA1 are pro-inflammatory in nature and act in a similar manner (not antagonistic manner as claimed) [23] in DSS-induced colitis using TRPA1 and TRPV1 knockout mice [27]. The association of TRP channels toward inflammation and immunogenic responses has been largely found to be positively regulated during the immune-physiology of the cellular and systemic responses. TRPA1 has been reported to contribute to the inflammation-induced pain and is also associated with experimental colitis in mice models [26–30]. Taken together, our data are in line with majority of reports showing TRPA1 as a pro-inflammatory regulator.

Inhibition of TRPA1 by its specific inhibitor reduces TCR- and ConA-driven mitogenic activation of T cells. This suggests that TRPA1 might be involved in the signaling pathways toward T-cell activation. Thus, inhibiting TRPA1 activity has profound inhibitory effects on CD25 and CD69 expression together with the secretion of signature effector cytokines such as TNF, IFNγ, and IL-2. TNF production is associated with several pro-inflammatory responses [31]. Additionally TNF is shown to be a major mediator of different inflammatory disease conditions like colitis and rheumatoid arthritis (RA) [32,33]. TRPA1 channel expression is found to be increased in peripheral blood leukocytes of RA patients and associated with pain [34]. Moreover, it has been shown that TRPA1 facilitates TNF-directed inflammatory responses in various pathophysiological conditions and blockade of TRPA1 receptors may be beneficial in reducing TNF-induced chronic pain [35,36]. Functional role of IFNγ as a signature Th1 cytokine is also implicated in pro-inflammatory responses and disease conditions like colitis [37]. However, there are some reports which suggest an anti-inflammatory role of IFNγ in the mouse model of colitis as well [38,39]. Apparently, these reports may indicate that these cytokines may differentially regulate inflammatory responses in different disease conditions. Interestingly, we have found that during *in vitro* TCR activation the induction of signature Th1 and pro-inflammatory cytokines like IL-2, IFNγ, and TNF could be down-regulated by the TRPA1 specific inhibitor, A-967079. TRPA1^−/−^ CD4^+^ T cells have been recently reported to produce higher IL-2 and IFNγ but not TNF during TCR stimulation [23]. This difference with our data could be due to difference in the type of cells used or a complicated consequence of TRPA1 knockdown triggering compensatory roles by other ion channels in knockout animals. Also T cells derived from colitis-induced animals can be very different from naïve T cells isolated from healthy individuals.

**Figure 9:**
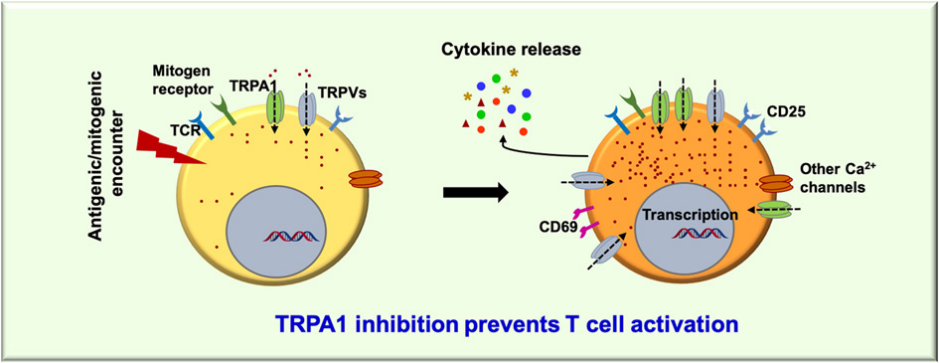
A proposed model for the expression and involvement of TRPA1 in T-cell activation Both percentage of T cells and level of TRPA1 per cell increases during T-cell activation. TRPA1 activation mediates Ca^2+^-influx to the cell. Inhibition of endogenous TRPA1 activity reduces the T-cell activation and release of certain cytokines. However, further investigation are needed to explore how TRPA1 regulates T-cell activation and cytokine release during T-cell activation process.

Moreover, ConA and TCR stimulated induction of T-cell activation markers like CD25 and CD69 were also found to be down-regulated in presence of A-967079. TRPA1 inhibition not only prevents T-cell activation in the general CD3^+^ T cells, but also in subsets of T cells like CD4^+^ and CD8^+^ T cells (data not shown). This role of TRPA1 in T-cell activation could be primarily due to its role in regulating intracellular Ca^2+^ levels in T cells. TRPA1 upon activation by its specific activator (AITC) was found to increase the Ca^2+^ levels, while two different inhibitors namely A-967079 and HC-030031 are able to reduce the intracellular Ca^2+^ levels. Therefore, it seems that during T-cell activation, TRPA1 becomes functional and shows augmented expression and executes immune-regulatory functions, whereas, inhibition of this channel inhibits T-cell activation. Recent report demonstrating the activation of TRPA1 by specific microRNA can also be relevant for T-cell activation [40]. While our current work confirms the functional expression of TRPA1 channel and its requirement toward T-cell activation, the involvement of this ion channel in T-cell activation process associated with inflammatory diseases needs further investigation (Figure 9).

In brief, our current findings demonstrated an *in vitro* T-cell activation directed functional expression and requirement of TRPA1 in T cells. This is in line with the earlier reports of inflammatory responses associated with the function of TRPA1 in various physiological systems. The current observation might have implication in the immunogenic and inflammatory role of T cell responses as well.

## Data Availability

All data generated during the present study are included in this published article.

## Abbreviations

AITC: allyl isothiocyanate
APC: antigen-presenting cell
BAPTA-AM: 1,2-bis(2-aminophenoxy)ethane-N,N,N′,N′-tetraacetic acid tetrakis (acetoxymethyl ester)
Ca^2+^: calcium ion
CD25: cluster of differentiation 25
CD69: cluster of differentiation 69
ConA: Concanavalin A
EGTA: ethylene glycol-bis(β-aminoethyl ether)-*N*,*N*,*N*′,*N*′-tetraacetic acid
ELISA: enzyme-linked immunosorbent assay
hPBMC: human peripheral blood mononuclear cell
IFNγ: interferon-γ
IL: interleukin
RA: rheumatoid arthritis
RT-PCR: reverse transcription polymerase chain reaction
TNF: tumor necrosis factor
TRPA1: transient receptor potential channel subfamily A member 1
TRPV: transient receptor potential cation channel subfamily vanilloid transient receptor potential Ankyrin1
